# The influence of exercise intensity on comorbid anxious behavior in psychiatric conditions

**DOI:** 10.1186/s12576-024-00930-7

**Published:** 2024-08-02

**Authors:** Dong-Joo Hwang, Tae-Kyung Kim

**Affiliations:** 1 Exercise Biochemistry Laboratory, Korea National Sport University, 1239, Yangjae-ro, Songpa-gu, Seoul, 05541 Korea; 2 Sport Science Institute, Korea National Sport University, 1239, Yangjae-ro, Songpa-gu, Seoul, 05541 Korea; 3 Department of Physical Education, Korea National Sport University, 1239, Yangjae-ro, Songpa-gu, Seoul, 05541 Korea

**Keywords:** Depression, Autism spectrum disorder, Exercise intensity, Anxiety-like behavior, Psychiatric condition

## Abstract

Many experts have extensively studied the potential of exercise as a treatment option for psychiatric conditions, including depression and autism spectrum disorder (ASD). Despite their core symptoms, these conditions exhibits comparable component traits, an anxiety. In this study, we explored the effect of exercise on behavioral abnormalities in psychiatric conditions, focusing on its intensity and emotional resilience. Shank3B knockout (KO^SED^) mice displaying self-injurious repetitive behavior and C57BL/6J mice, susceptible to stress as ASD and depression model, respectively, were subjected to moderate-intensity exercise (ME) for 2 weeks. ME mitigated the core symptoms (excessive grooming traits and behavioral despair) but did not exert a significant anxiolytic effect. Notably, exercise intensity has emerged as a critical determinant of its efficacy, as evidenced by a lower ventilation threshold and anxiolytic effect mediated by low-intensity exercise. The findings substantiate the notion that exercise is promising as a disease-modifying treatment, but intensity matters for emotional resilience.

**Graphical Abstract:**

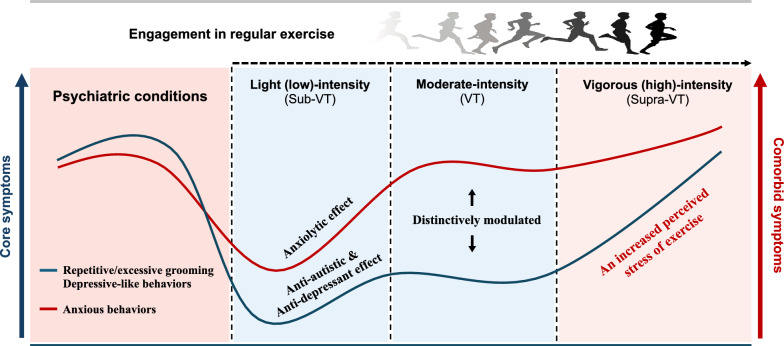

**Supplementary Information:**

The online version contains supplementary material available at 10.1186/s12576-024-00930-7.

## Background

Psychiatric disorders encompassing conditions such as depression, anxiety, and autism (referred to as autism spectrum disorders (ASD)) pose significant challenges to global health, affecting individuals across diverse age ranges and socioeconomic backgrounds [1, 2]. Despite extensive efforts, substantial gaps remain in understanding the behavioral outcomes of psychiatric conditions owing to their genetic and behavioral complexity and heterogeneity, leading to difficulties in early diagnosis, prevention, and treatment. ASD symptoms vary widely among individuals but are typically characterized by impairments in reciprocal social interactions and repetitive and stereotyped interests and behaviors [3]. There is strong evidence supporting that genetic (mutations in genes and chromosomal abnormalities) and environmental influences (e.g., maternal exposure to certain medications during pregnancy and prenatal exposure to infections or toxins) have implications for ASD. Interestingly, individuals with ASD have an increased risk of depression and anxiety compared with typically developing populations [3, 4], and it had been reported that depressive symptoms emerges as a primary concern among adults with autism prior to being diagnosed with ASD [5, 6]. Major depressive disorder (MDD) is a heterogeneous psychiatric disorder involving an interplay of diverse etiological factors. They exhibit a range of behavioral alterations, including loss of interest and pleasure, persistent feelings of sadness, and frequent anxiogenic behaviors [7]. ASD and MDD are complex conditions that present distinct core symptoms; however, these two psychiatric conditions exhibit comparable component traits, an anxiety. The presence of anxiety exacerbates core symptoms of ASD and MDD across development and diminishes the probability of attaining remission in individuals with MDD [8, 9]. Therefore, elucidating the intricate interaction between anxiety, ASD, and MDD is imperative for enhancing diagnostic accuracy, therapeutic interventions, and implementing targeted management strategies.

Regardless of genetic and environmental factors, some modifiable factors can be targeted by therapeutic approaches to prevent or alleviate the symptoms of ASD and MDD. Although conventional treatment approaches involving pharmacotherapy and psychotherapy focus on managing symptoms characterized by disturbances in thoughts, emotions, and behavioral deficits, disease modification has gained attention as a promising avenue for treatment [10, 11]. Medication helps many; however, the high failure rate and potential side effects associated with the interventions have prompted researchers to explore alternative or adjunctive treatment options that are less disruptive, time-consuming, and expensive [12]. Emerging evidence suggests that lifestyle changes, including exercise, stress management, and a healthy diet, may offer promising disease-modifying benefits for psychiatric conditions, and researchers have been making progress in understanding whether and how it works.

Physical exercise has long been recognized for its positive impact on physical health, particularly in preventing and managing metabolic disorders, including diabetes, obesity, and cardiovascular disease. More recently, attention has been focused on the potential effects of exercise as a non-pharmacological and non-invasive intervention for mental health problems [13–15]. The idea that exercise can exert profound structural and functional benefits in psychiatric conditions is supported by several studies of human and animal models. Multiple studies have demonstrated that exercise improves behavioral deficits associated with psychiatric conditions, including MDD, anxiety, and cognitive function [13, 15]. Moreover, exercise modulates various neurological mechanisms, including improved neuroplasticity, neuroinflammation, the immune system, and the regulation of neurotransmitter release, which contribute to the pathophysiology of psychiatric conditions. The evidence is compelling; nevertheless, the effectiveness of exercise as a disease-modifying treatment for psychiatric conditions has not been definitively established, and some reports from studies with different modalities such as exercise intensity have yielded inconclusive or conflicting results [11, 15, 16]. Therefore, further investigation is necessary to elucidate the efficacy of exercise in psychiatric conditions and, subsequently, the optimal exercise modalities depending on the intensity. We aimed to explore the effect of exercise on behavior abnormalities in models of psychiatric disorders, focusing on exercise intensity and its implications for clinical practice. Our study provides strong evidence of the exercise benefits on core and comorbid symptoms of ASD and MDD models and how behavioral deficits are distinctively modulated by exercise depending on intensity.

## Materials and methods

### Animals

Strain-matched Shank3B knockout (KO) mice (Shank3B^−/−^) displaying excessive grooming and self-injurious repetitive behaviors and naïve C57BL/6J mice susceptible to stress (SUS^SED^) (see the chronic restraint stress section) were used to explore the efficacy of exercise in ASD and depression model. For ASD, male mutant mice (strain #017688) carrying a neomycin resistance (neo) cassette replacing the PDZ domain (exon 13–16) of the SH3/ankyrin domain 3 (*Shank3*) gene were purchased from Jackson Laboratory and crossed with wild-type (WT) littermates. Experimental homozygous Shank3B^−/−^ (Shank3B^KO^) and WT mice (13–14 weeks old, male) were obtained via heterozygous mating. The primers used for genotyping were designed against the following primers: for the WT and mutant allele: 5’-TGA CAT AAT CGC TGG CAA AG-3’ and 5’-GCT ATA CGA AGT TAT GTC GAC TAG G-3’; for the common allele: 5’-GAG ACT GAT CAG CGC AGT TG-3’. For depressive disorder, eight-week-old male C57BL/6J mice were purchased from Daehan Bio-Link (Eumseong, Chungbuk, Korea). All mice were housed in a controlled pathogen-free environment (12 h:12-h light–dark cycle, 20 ± 2 °C, 50–55% humidity) and given ad libitum access to food (SAFE® R04-10, Aston Pharma, England) and water. All procedures were performed in accordance with the animal care guidelines of the Animal Ethics Committee of Korea National Sport University (KNSU) and approved by the Institutional Animal Care and Use Committee of KNSU (approval numbers: KNSU-IACUC-2022-08 and KNSU-IACUC-2023-02).

### Restraint stress

The chronic restraint stress (RST) was performed as previously described [17, 18]. Each mouse was placed in a ventilated 50 ml polyethylene tube to maintain immobilization for 2 h daily from 7 p.m. to 9 p.m. (during dark cycle); this procedure was repeated for 14 days. After chronic RST, the mice were transferred to their home cages for a standard chow diet and water.

Identification of mice susceptible and resilient to stress was performed based on the immobility time (s) of individual animals, measured using the forced swim test (FST) and tail suspension test (TST) after the chronic RST treatment. For classification, individual values of behavior test were converted into z-score (*z* = (*x*-*μ*)/*σ*) that measures the distance between a data point and the mean using standard deviations and then analyzed by K-means clustering method, an unsupervised machine learning algorithm used for clustering data points into clusters based on their similarity. Data points that belong to the cluster whose mean (centroid) is closest to the dataset of sedentary mice were labeled “resilient”, whereas those with another cluster (distant from the dataset of sedentary) were labeled “susceptible” [17–19].

### Behavior assessment

Behavior assessment was performed as previously described. All tests were video-recorded using a camera or an automated tracking system (SMART, Panlab, Barcelona, Spain). The behavioral testing room was lit with approximately dimmable panel light (20–40 lx) for the grooming and open field tests (OFT). All the mice in their home cages were transported to a behavior testing room for habituation to the testing environment before commencing behavior testing. All parts of the apparatus were cleaned with 70% ethanol to eliminate the effect of smell on the spontaneous activity of the next subject.

### Grooming behavior

Young adult male mice (15–16 weeks old) were used for the self-grooming test [20, 21]. After placing the habituated (20 min) individual mouse into the acrylic cage (20 × 28 × 13 cm), video recording was initiated and lasted for 10 min under low-light (20 lx) illumination. Grooming behavior was observed at a specific time point within the dark cycle (from 11 p.m. to 12 p.m.), and all the sequences of face wiping, scratching/rubbing of the head, ears, and tail, and whole-body grooming with paws and tongue were manually counted. Scoring (time and count) of the video-recorded grooming behaviors of the mice was conducted by an experienced observer blinded to the experimental conditions.

### Tail suspension test

TST was conducted as previously described [17, 22]. The mice were suspended in adhesive tape applied to the tail tip 70 cm above the ground. During the 6-min recording session, the cumulative immobility time for the last 5 min (after the initial 1 min of the excessive struggling period), indicative of depressive-like behavior, was measured. It was defined as immobility when the mice stopped struggling and hung immobile.

### Forced swim test

In FST, mice were individually placed in a transparent cylinder (15 cm in diameter × 27 cm in height) filled with tap water (23–25 °C) for 6 min, and all the procedure was video-recorded. Only the last 5 min of the test were analyzed, and the cumulative immobility time with only the minimal movement necessary to keep the animal’s head from being submerged under the water was counted. Escape behaviors, including swimming with horizontal movement and climbing with upward movement of the forepaws, were excluded from the analysis [17, 22].

### Open field test

As indications of anxiogenic behavior, the level of general activity and locomotion of mice exposed to large, brightly bit, open, and unfamiliar environments was measured using zone analysis [23]. Mice were placed in an open field apparatus (45 × 45 × 40 cm) with a floor divided into 25 squares (the center nine squares as the center zone and the remaining 16 squares as the peripheral zone) and allowed to explore freely for 5 min while being recorded by a camera. The path was analyzed using an automated tracking system (SMART, Panlab, Barcelona, Spain) for the following parameters: distance traveled, and time spent in the center and peripheral zones.

### Elevated plus maze

In EPM, mice were placed at the center zone of a plus ( +) shaped maze positioned 600 mm above the floor, illuminated evenly at 20 lx, and consisted of four arms, with two open arms without walls and two enclosed arms by 350 mm length × 50 mm width × 200 mm height walls), facing the open arm opposite to where the experimenters are [24]. Then, mice were allowed to freely explore the maze for 5 min, and the path was analyzed using an automated tracking system (SMART, Panlab, Barcelona, Spain) for the following parameters: time spent in the open, closed, and center zone (%) and the number of arm entries made by mice onto each arm. At the end of the test, the mice were returned to their home cage.

### Incremental exercise test

An incremental running test was performed to measure the ventilation threshold (VT) measurement as an indicator of exercise intensity and capacity, as previously described [25, 26]. After three days of adaptation periods comprising 10 min of gradual running at 0–10 m/min, the VT level was determined. The incremental running test was initiated by 20-min rest and followed by running at 4.8 m/min; the treadmill speed was progressively increased by 2.4 m/min every 2 min at a fixed gradient of 5° inclination until exhaustion (a state at which the mice could not keep their pace and remained at the back of the treadmill despite repeated agitation). The inflection point (VT) was estimated using the V-slope method, which plots VO_2_ and VCO_2_ on an X- and Y-axis grid and finds the point at which the increase in VCO_2_ is greater than the increase in VO_2_ by piecewise regression and two-segmented linearity [26, 27].

### Exercise training

Mice subjected to exercise underwent training on a motorized rodent treadmill (NG, SciTech Korea Inc., Korea). Following habituation to the treadmill apparatus for 5 consecutive days at 6–10 m/min for 10 min at a gradient of 0° inclination, the mice were assigned to continuous exercise training at a speed of 11–13 m/min for 40 min/day (including initial 10-min rest), 5 days/week for 2 weeks, based on the results of incremental exercise test and previous findings, which is a moderate exercise intensity ranging from approximately 45 to 65% of VO_2_max or VO2peak [27–29]. Non-exercised mice in the susceptible group (SUS^SED^) received no treatment to ensure they remained in the same environment as the exercised group during the exercise intervention, thereby excluding resulting from environmental exposure.

As an index of exercise intensity, the VT appeared at a running speed of approximately 6.5 m/min and 8.8 m/min for SUS^SED^ and KO^SED^ mice, respectively (Fig. 3). Thus, the running speed for exercise training of SUS^SED^ and KO^SED^ mice was categorized at 6–7 m/min (sub-VT or low-intensity, LE, averaging 175.88 m per day), 11–13 m/min (VT or moderate-intensity, ME, averaging 346.08 m per day), and 16–18 m/min (supra-VT or high-intensity, HE, averaging 489.59 m per day) for each exercised group.

### A single bout of exercise and lactate measurement

Mice were subjected to treadmill exercise at a designated speed for 30 min, which was a point of resting state or below (sub-VT) and above the VT (supra-VT), as measured by the graded exercise test mentioned above. The mice were stimulated with a gentle nudge when they could not keep pace. Blood collected by cardiac puncture was immediately analyzed using an automatic lactate analyzer (YSI 1500, Yellow Springs Instruments Co., Inc., USA).

### Enzyme-linked immunosorbent assay

Serum corticosterone (CORT) levels were measured using CORT ELISA assay kit (Enzo Life Sciences, ADI-900-097) according to manufacturer’s instruction. Blood was collected from the abdominal aortas of mice, and serum was obtained by centrifuging at 3000 rpm for 15 min and then stored at 80 °C until use. Briefly, serum sample (10ul) from each group was diluted with an equal volume of a steroid-displacement reagent at a ratio of 1:40 and mixed on a 96-well plate. The mixture was then incubated on a plate shaker (300 rpm) for 2 h at room temperature (RT). After incubation, the mixtures were discarded, and the plate was washed 3 times with washing buffer, followed by serial standard protocol. The reaction was stopped using the stop solution provided, and the absorbance was measured at 405 nm using a microplate reader (HIDEX, Turku, Finland). The concentration of CORT was calculated from the standard curve and analyzed using an online platform for analyzing CORT ELISA results of Enzo Life Sciences’ product (http://www.myassays.com).

### Statistics

Statistical analyses were performed using GraphPad Prism 8 software (GraphPad Prism Inc., CA, USA) and SPSS Statistics 25 software (IBM SPSS Statistics, NY, USA). The normality of data was assessed using the Shapiro–Wilk tests, while statistical significance was analyzed using the Mann–Whitney U-test for two groups and one-way analysis of variance (ANOVA) followed by a Bonferroni post hoc test for multiple comparisons. K-means clustering was used to classify the behavioral features of individually stressed animals measured by behavioral tests into two clusters (susceptible to stress and resilient to stress), maximizing the between-cluster distance and minimizing the within-cluster distance. Data are expressed as mean ± standard error (SEM), and statistical significance was tested at *p* < 0.05.

## Results

### ME ameliorates autistic behavior in ASD model but not anxiety-like behavior

**Fig. 1:**
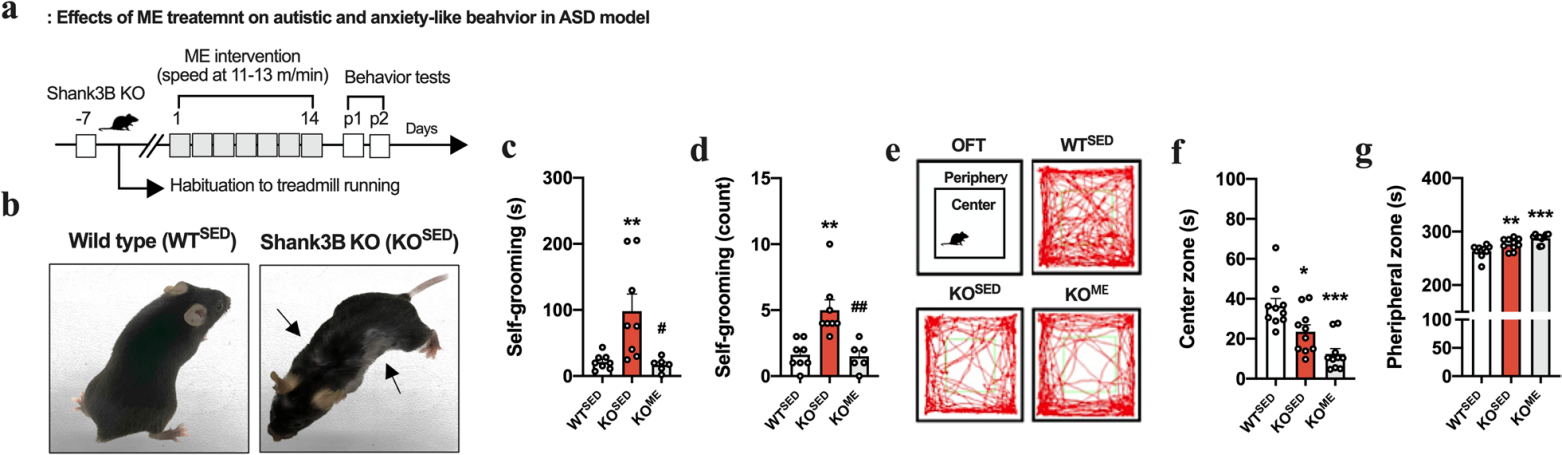
Impact of exercise on behavioral abnormalities in genetically modified (Shank3B KO) ASD model. **a** Experimental design. **b** Representative images of skin lesions observed in WT^SED^ and KO^SED^ (Shank3B^KO^) models. **c**, **d** The self-grooming duration and count in grooming behavior test (*n* = 6–8 mice/group). **e** Representative tracking images of movement in OFT. **f**, **g** Time spent in center and peripheral zones in OFT (*n* = 10 mice/group). Data are presented as mean ± SEM. Each circle represents individual data points. *p < .05, **p < .01, ***p < .001 versus WT^SED^, ^#^*p* < .05, ^##^*p* < .01 versus KO^SED^

To investigate whether ME attenuates behavioral abnormalities in KO^SED^ mice, we performed a self-grooming test for repetitive behavior and an OFT for anxiety-like behavior (Fig. 1). After 2 weeks of ME treatment, the self-injurious excessive grooming behavior observed in KO^SED^ mice that causes a pronounced skin-lesion was significantly reduced (*F*_(2,19)_ = 7.56, *p* < 0.01 in self-grooming duration and *F*_(2,19)_ = 12.0, *p* < 0.001 in self-grooming count; Fig. 1b–d). Unexpectedly, however, in the zone analysis of the OFT, ME did not affect or exacerbate the anxiety-like behavior (aversion to open space) of KO^SED^ mice, as shown by the significantly decreased time spent in the center zone (*F*_(2,27)_ = 13.3, *p* < 0.001) and increased time spent in the peripheral zone (*F*_(2,27)_ = 13.3, *p* < 0.001) (Fig. 1e–g).

### ME attenuates behavior despair in susceptible mice to stress but not anxiety-like behavior

**Fig. 2:**
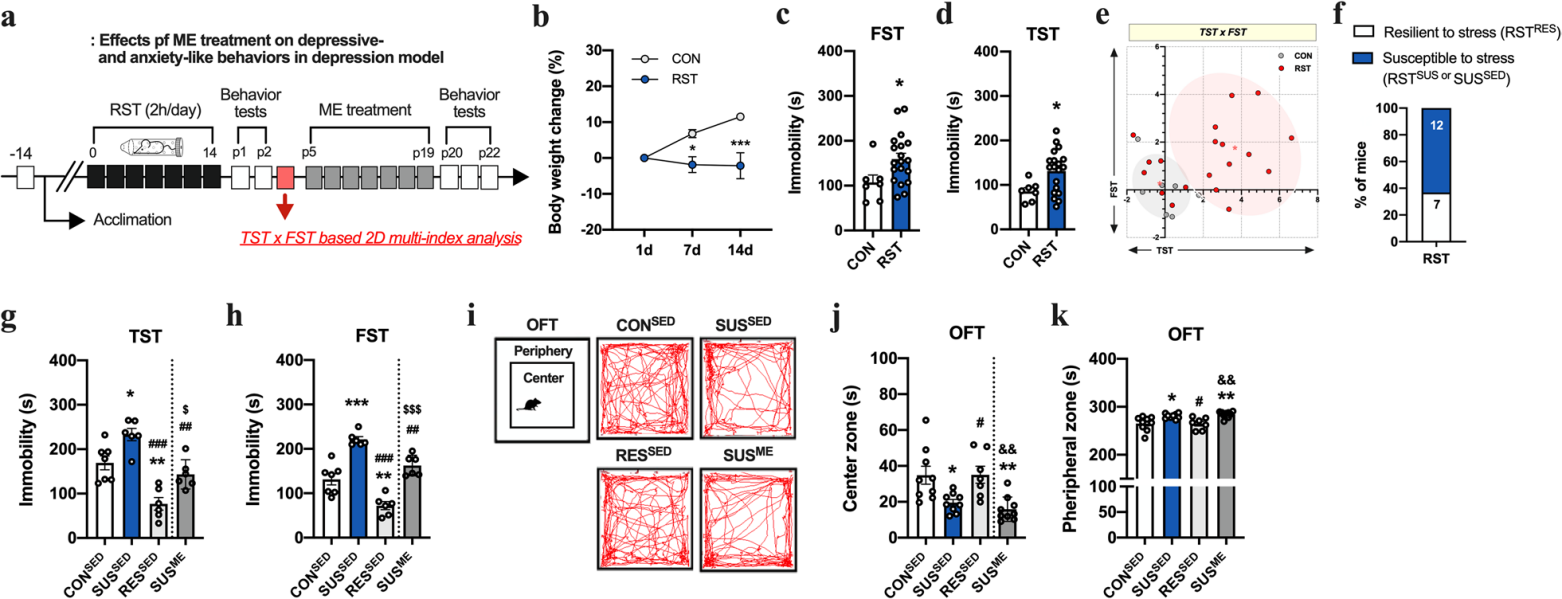
Identification of mice susceptible to stress and the impact of exercise on depressive- and anxiety-like behaviors in a chronic RST-induced depression model. **a** Experimental design. **b** Changes (%) in body weight in mice treated with chronic RST. **c**, **d** Immobility time in TST and FST (n = 7 and 19 mice in CON and RST groups). **e**, **f** Clustering of individual mice in the TST and FST matrix plotting with z-scores and proportion (%) of each group in the cluster (63.16% for susceptible and 36.84% for resilient mice to stress). **g–h** Immobility time in TST and FST (n = 6–7 mice in CON^SED^, SUS^SED^, RES^SED^, SUS^ME^ groups). **i** Representative tracking images of movement in OFT. **j-k** Time spent in center and peripheral zone in OFT (n = 9 mice/group). Data are presented as mean ± SEM. Each circle represents individual data points. *p < .05, **p < .01, ***p < .001 versus CON^SED^, ^#^*p* < .05, ^##^*p* < .01, ^###^*p* < 0.001 versus SUS^SED^. ^$^*p* < .05, ^$$$^*p* < .001 versus RES^SED^

The TST and FST were used to assess depression-like behavior (Fig. 2). As expected, mice exposed to the chronic RST showed significantly increased immobility time (TST: *p* < 0.05; FST: *p* < 0.05), with progressive suppression of body weight gain (*F*_(2,36)_ = 6.068, *p* < 0.01; Fig. 2b–d). Furthermore, K-means clustering analysis of individual animals in the TST × FST matrix indicated that approximately 63% (12/19) of the chronic RST-treated mice were susceptible to stress (Fig. 2e-f). After 2 weeks of ME treatment, we examined whether ME attenuated the severe behavioral despair observed in SUS^SED^ mice. SUS^SED^ mice treated with ME exhibited decreased immobility time in the TST (*F*_(3,21)_ = 19.2, *p* < 0.001) and FST (*F*_(3,21)_ = 37.4, *p* < 0.001), but not to the same extent as sedentary (CON^SED^) and RES^SUS^ mice (Fig. 2g–h). Unexpectedly, ME did not affect or exacerbate the anxiety-like behavior of SUS^SED^ mice, as shown by the significantly decreased time spent in the center zone (*F*_(3, 30)_ = 7.72, *p* < 0.01) and increased time spent in the peripheral zone (*F*_(3,30)_ = 7.72, *p* < 0.001) (Fig. 2i–k).

### Differences in intrinsic exercise capacity due to psychiatric illness

**Fig. 3:**
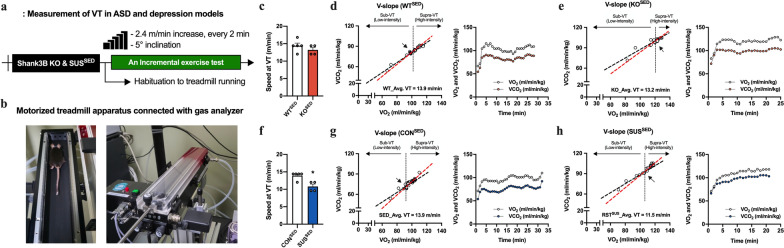
The measurement of ventilation threshold (VT) in KO^SED^ and SUS^SED^ mice. **a** Experimental design. **b** Motorized treadmill apparatus connected to gas analyzer. **c** Mean speed at VT from WT^SED^ and KO^SED^ mice (*n* = 4–5 mice/group). Determination of the inflection point by V-slope method and transition of VO_2_ and VCO_2_ values measured by an incremental exercise test in **d** WT^SED^, **e** KO^SED^, **g** CON^SED^, and **h** SUS^SED^ mice. **f** Mean speed at VT from SED and SUS^SED^ mice (*n* = 4–5 mice/group). Black and red dashed lines indicate linear curves estimated from regression analysis. Sub-VT and supra-VT were defined as low- or light-intensity and high-intensity, respectively. Data are presented as mean ± SEM. Each circle represents individual data points. *p < .05 versus CON^SED^

VT is the point at which the body’s aerobic energy production becomes insufficient to meet the demand for exercise intensity, leading to an increased reliance on anaerobic metabolism. This can help individuals exercise within the appropriate intensity ranges. To determine the VT in KO^SED^ and SUS^SED^ mice, we performed an incremental running test using an indirect calorimetric system equipped with a treadmill (Fig. 3). With progressively increasing volume of O_2_ consumption and CO_2_ production, the VT was defined as speed at 13.9, 13.2, 13.9, and 11.5 m/min in WT^SED^ (Fig. 3c, d), KO^SED^ (Fig. 3c, e), CON^SED^ (Fig. 3f, g), and SUS^SED^ (Fig. 3f, h) mice, respectively. No significant differences in running speed during VT were observed between KO^SED^ mice (Fig. 3c), whereas SUS^SED^ mice had relatively lower levels of VT than CON^SED^ mice (*p* < 0.05; Fig. 3f). Notably, KO^SED^ and SUS^SED^ mice subjected to supra-VT showed higher blood lactate concentrations than the resting and sub-VT mice (*F*_(2,9)_ = 14.6, *p* < 0.01 in SUS^SED^ and *F*_(2,6)_ = 28.8, *p* < 0.001 in KO^SED^; Supple Fig. 1a, b), indicating that exercise-inducible physiological stress was lower at sub-VT intensity (LE), while quite stressful by other exercise intensities above VT.

### LE possibly reversed autistic- and anxiety-like behaviors in ASD model

**Fig. 4:**
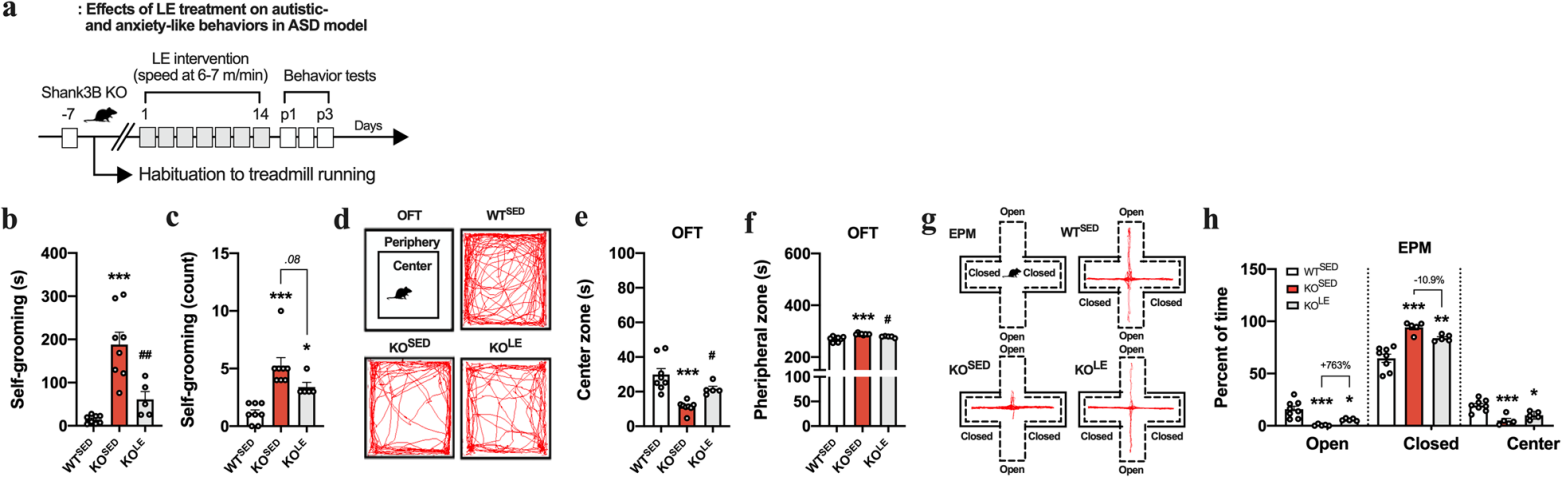
LE ameliorate the repetitive self-injurious grooming behavior and comorbid anxious behavior in ASD model. **a** Experimental design. **b**, **c** The self-grooming duration and count in grooming behavior test (*n* = 5–8 mice/group). **d** Representative tracking images of movement in OFT. **e**, **f** Time spent in center and peripheral zones in OFT (*n* = 5–8 mice/group). **g** Representative tracking images of movement in EPM **h** Time spent in closed arm, open arm, and central zone in EPM (*n* = 5–8 mice/group). Data are presented as mean ± SEM. Each circle represents individual data points. *p < .05, ** *p* < .01, ***p < .001 versus WT^SED^, ^#^*p* < .05, ^##^*p* < .01 versus KO^SED^

Based on the VT level measured by incremental exercise test for KO^SED^ mice, we tested whether engagement in regular exercise below VT (LE) could potentially attenuate both autistic and anxiety-like behaviors simultaneously (Fig. 4). Notably, KO^SED^ mice subjected to exercise training below VT (Low-intensity) showed less severe self-injurious grooming behavior, as evidenced by decreased self-grooming duration (*F*_(2,18)_ = 21.3, *p* < 0.001; Fig. 4b, c). Consistent with the findings related to core symptoms of ASD model, KO^LE^ mice exhibited an increased time spent in center zone (*F*_(2,18)_ = 15.8, *p* < 0.001) and the decreased time spent in peripheral zone during OFT (*F*_(2,18)_ = 15.8, *p* < 0.001) (Fig. 4e, f). In EPM, KO^LE^ mice tended to spend more time in the open area (763% increase in KO^LE^ compared to KO^SED^) and less time in closed area (10.9% decrease in KO^LE^ compared to KO^SED^), although the difference was not statistically significant (Fig. 4g, h). Considering the EPM’s higher anxiogenic nature compared to OFT, as suggested by previous studies, these results indicate that regular exercise engagement could possibly attenuate both autistic behavior and comorbid anxiety-like behavior, with distinctive effects depending on intensity.

### LE mitigates depressive- and anxiety-like behaviors in susceptible mice to stress

**Fig. 5:**
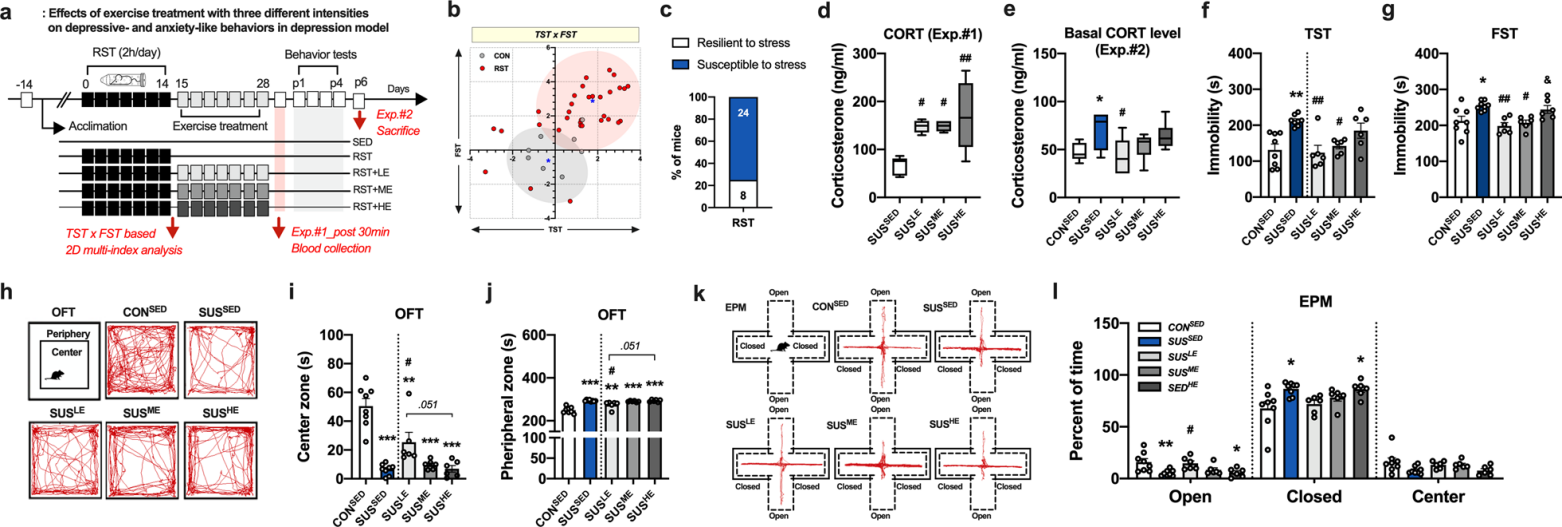
Effect of different exercise intensities on depressive- and anxiety-like behaviors in susceptible mice to stress. **a** Experimental design. **b**, **c** Clustering of individual mice in the TST and FST matrix plotting with z-scores and proportion (%) of each group in the cluster (75% for susceptible and 25% for resilient mice to stress). **d** Exercise intensity-dependent CORT levels immediately after exercise treatment (Exp.#1). **e** Basal CORT levels after 2 weeks of exercise treatment with different intensities in indicated groups (Exp.#2). **f**, **g** Immobility time in TST and FST (*n* = 6–8 mice in CON^SED^, SUS^SED^, SUS^LE^, SUS^ME^, SUS^HE^ groups). **h** Representative tracking images of movement in OFT. **i**, **j** Time spent in center and peripheral zone in OFT (*n* = 6 mice/group). **k** Representative tracking images of movement in EPM. **l** Time spent in closed arm, open arm, and central zone in EPM (*n* = 6–8 mice/group). Data are presented as mean ± SEM. Each circle represents individual data points. *p < .05, **p < .01, ***p < .001 versus CON^SED^, ^#^*p* < .05, ^##^*p* < .01, ^###^*p* < .001versus SUS^SED^, ^$^*p* < .05 versus SUS^LE^

As mentioned earlier, we investigated whether the 2 weeks of exercise training with different intensities (low-, moderate-, and high-intensity) regulates behavior deficits induced by chronic RST (Fig. 5). First, K-means clustering analysis of individual animals in the TST × FST matrix indicated that approximately 75% (24/32) of the chronic RST-treated mice were susceptible to stress (Fig. 5b, c). Prior to behavior tests, we measured the physiological and psychological stress load evoked by exercise treatment with different intensities following chronic RST treatment. The results showed that all the exercise regimes led to an increased CORT response immediately after exercise in susceptible mice to stress, with a tendency of a much higher increase in intense exercise group (SUS^HE^) (*F*_(3,16)_ = 7.27, *p* < 0.01, Fig. 5d). In addition, the significantly increased basal levels of CORT (5 days after last exercise session) in SUS^SED^ mice was reduced by LE, but not significant in SUS^ME^ and SUS^HE^ mice (*F*_(4,28)_ = 3.95, *p* < 0.05, Fig. 5e). Interestingly, exercise training at the level of VT (ME) and/or below VT (LE) reversed the depressive-like behavior, as evidenced by decreased immobility time in both the TST and FST (*F*_(4,29)_ = 6.23, *p* < 0.001 in TST and *F*_(4,29)_ = 6.41, *p* < 0.001 in FST; Fig. 5f, g). However, exercise training above VT (high-intensity) did not alter the immobility time in either the TST or FST (Fig. 5f, g). To further investigate whether the exacerbated anxiety-like behavior observed in SUS^SED^ mice subjected to ME (refer to Fig. 2j, k) resulted from adverse aspects of vigorous exercise intensity, we tested the impact of different exercise intensities in susceptible mice to stress (Fig. 5). Notably, after 2 weeks of exercise training with different intensities, exacerbated anxiety-like behaviors demonstrated by both OFT and EPM in susceptible mice to stress (SUS^SED^) were attenuated in SUS^LE^ mice, but not in SUS^ME^ and SUS^HE^ mice (*F*_(4,29)_ = 24.1, *p* < 0.001 in time spent in center zone of OFT; *F*_(4,29)_ = 24.1, *p* < 0.001 in time spent in peripheral zone of OFT; *F*_(4,29)_ = 5.23, *p* < 0.001 in percent of time in open area of EPM; *F*_(4,29)_ = 4.14, *p* < 0.01 in percent of time in closed area of EPM; Fig. 5h–l).

## Discussion

Although distinct, patients with ASD and MDD share comparable component traits that require further exploration. Individuals diagnosed with ASD or MDD often encounter difficulties in emotional resilience, including anxiety-like behaviors, that require careful evaluation during diagnosis and treatment. Remarkably, experts have suggested that engaging in regular exercise could have therapeutic potential for psychiatric conditions. Here, we explored the efficacy of exercise in an animal model of ASD and MDD and substantiated the necessity of an optimal exercise modality. Our results revealed that ME, recognized for its therapeutic effects on brain plasticity, effectively modulated the progression of autistic and depressive-like behaviors in Shank3B-deficient and chronic RST-treated mice. This suggest its potential as a treatment with disease-modifying capabilities. Nevertheless, ME intervention exhibited no noticeable effect on comparable component trait, an anxiety-like behavior in either psychiatric condition, which could be attributed to differential metabolic characteristics and physiological or psychological stress evoked by a more intense exercise regimen than originally expected.

Interestingly, however, LE not only alleviated core symptoms but also mitigated comorbid anxiety-like behavior, in both ASD and MDD models. These results highlight that ME may ameliorate the core symptoms of ASD and MDD models, However, its intensity affects anxiety, a comorbid symptom, suggesting that the heterogeneous psychiatric conditions with various subtypes, including autistic-, depressive-, and anxious-behaviors, are associated with distinct neurobiological basis and LE than moderate- or vigorous-intensity might be a more suitable therapeutic option for psychiatric illness.

According to the previous studies encompassing ASD and MDD, the genetic disruption of Shank3B leads to altered synaptic dysfunction, which contributes to the development of compulsive/repetitive behaviors resembling the cardinal features of ASD [20, 21, 23], and exposure to chronic stress precipitates the onset of psychiatric disorders, thereby heightening the propensity for developing depressive- and anxiety-like phenotypes [17, 19]. In the present study, it is noteworthy that at 4 months old, KO^SED^ mice developed pronounced skin lesions along with self-injurious excessive grooming behaviors compared to age-matched WT mice, indicating its substantial relevance as an ASD model (Fig. 1). Furthermore, after exposure to the chronic RST, approximately 63% (12/19) of individual RST-treated mice, whose stress vulnerability was assessed by clustering algorithm based on the TST and FST to provide insights into the biology of variations in stress reactivity and develop successful coping strategies for depression [18, 19, 30], were susceptible to stress (SUS^SED^) with severe depressive symptoms (Fig. 2). On the other hand, our findings revealed that, compared with sedentary mice, ASD and MDD models subjected to 2 weeks of ME exhibited a reversal psychotropic and antidepressant effect, manifested as less severe self-injurious grooming traits and behavioral despair, without any problems with their circadian rhythm. Along with previous findings supporting the possibility of exercise-induced neuroprotection through neuroplastic changes in the brain [29, 31], this finding may also reflect the therapeutic potential of exercise as a coping strategy in psychiatric conditions.

Since ASD and MDD models are distinct but exhibit comparable component traits such as anxiety, which can exacerbates core symptoms of ASD and MDD across development and diminishes the probability of attaining remission in individuals with MDD [8, 9], examining comorbid anxiogenic behavior represented by exploring new environments and aversion to exposed space could provide valuable insights into the distinct behavioral abnormalities and shared characteristics between these two psychiatric conditions. Thus, we examined whether ME had the same anxiolytic traits and reversal effects in both psychiatric conditions. Similar to the findings above, we observed severe anxiety-like behaviors in both KO^SED^ and SUS^SED^ mice. However, we did not detect a significant anxiolytic effect of ME. This finding may be surprising, as it contradicts the conventional expectation of exercise-inducible anxiolytic effects. It implies that exercise may induce some degree of aberrant neural processing, either exacerbating the perceptions of stress and anxiety or not altering them.

Currently, we lack a definitive explanation for the discrepancy in the ME-mediated reversal effect between core symptoms and anxiety-like behaviors. One possible explanation is that the optimal exercise intensity for psychiatric conditions could lead to phenotypic variance. The notion that exercise tries to break us, but its intensity or duration matter for emotional resilience has been consistently emphasized [15, 32–34]. Therefore, it is particularly significant to navigate the exercise intensity-dependent antidepressant and anxiolytic potentials upon consideration of a notable gap in existing researches. Previous studies investigating the anxiolytic effects of exercise in rodents have primarily utilized stress-free (VWR) and/or treadmill exercise paradigm, and in many cases, mice exercised during the same period of stress exposure, which may confound the interpretation of results. (notably, there is a lack of exercise-related investigation in the ASD model) [13, 14, 35–37]. Furthermore, while Otsuka et al. (2016) highlighted the crucial role of lower exercise intensity in determining the manifestation of anxiolytic effects, the exercise intensity employed in the study, based on the lactate threshold of rodents in a normal state, was not carefully considered in relation to the distinct requirements of individuals with psychological defects [34].

Interestingly, SUS^SED^ mice exhibited a relatively lower level of VT as an index of exercise capacity compared to sedentary mice but not in KO^SED^ mice. In previous studies [27, 29, 38], low- or light-intensity exercise was categorized at intensity below VT; however, the running speed of ME (11.5 m/min) in SUS^ME^ and KO^ME^ mice was slightly higher than the estimated one at Sub-VT (approximately 6.5 m/min and 8.8 m/min for SUS^SED^ and KO^SED^, respectively; Fig. 3). Therefore, the phenotypic variance in the anxiolytic effect of ME may be attributed to the physiological or psychological stress evoked by a more intense exercise regimen than originally expected. Accordingly, based on the VT level demonstrated by incremental exercise test (Fig. 3), we examined the anxiolytic effect of different exercise intensities (low-, moderate-, and high-intensity) separately in KO^SED^ and SUS^SED^ mice. Interestingly, as expected, regular LE engagement rescued the self-injurious grooming behavior and comorbid anxiety-like behavior in OFT. This anxiolytic effect of LE was also observed in an EPM, although not statistically significant, but with a decreased tendency, which might be due to EPM’s higher anxiogenic nature that stem from aversion to elevated and open area compared to OFT [39, 40].

Anxious depression is both prevalent and clinically significant, with estimates indicating that 40–50% of individuals diagnosed with MDD also exhibit comorbid anxiogenic traits [41, 42]. Consistent with the results from the ASD model, only LE (Sub-VT), but not moderate- and high-intensity exercise, mitigated the exacerbated depressive- and comorbid anxiety-like behaviors induced by chronic RST treatment, with relatively lower basal levels of CORT. This implies that an individual mouse might have a more heightened perception of exercise-inducible stresses compared to naïve mice, which might explain why ME led to phenotypic variance related to comparable component trait (anxiety-like behaviors) shared by ASD and MDD models (Fig. 1 and 2). Antidepressant and anxiolytic potential of exercise is well documented [31, 43]; however, it appears imperative to acknowledge that the heterogeneous psychiatric conditions with various subtypes, including autistic-, depressive-, and anxious-behaviors, are associated with distinct neurobiological basis [42] and exercise intensity emerges as a determinant factor, especially in comorbid anxiety-related behaviors.

The current study had several limitations. First, given the behavioral complexity and heterogeneity of psychiatric conditions, the molecular mechanisms underlying the therapeutic effects of exercise and its intensity-dependent variance should be investigated to gain a deeper understanding. Second, we were able to conduct the exercise training with high (vigorous)-intensity in the ASD model due to the issues related to the genetic ASD model breeding. Thus, extended follow-up research is warranted to ascertain whether HE can also modify anxiety-related phenotypes in ASD models. Third, exercise duration (> 2 weeks) cannot be excluded as potential contributing factor to the anxiolytic effect of ME, considering the nature of regular long-term exercise, which ultimately leads to positive changes in physical and psychological capabilities. Nevertheless, according to our research findings, it is evident that engaging in LE for no more than 2 weeks clearly alleviate anxiety-like behavior. Lastly, exercise benefits in stress-resilient mice is provided which could provide informative insights. However, as evident from the literature, studies targeting the therapeutic effects of exercise in stress-resilient animals are exceedingly scarce. Previous findings indicate that the efficacy of antidepressants, such as Fluoxetine, is behaviorally minimal in less stress-sensitive individuals, which may extend to exercise interventions [44], supporting the notion that the response to exercise in stress-resilient mice might be minimal due to their already reduced depressive- and anxiety-like behavior levels. Exploring this aspect in future research could nevertheless be valuable in further understanding the nuances of stress resilience and the potential benefits of exercise interventions.

Collectively, our results demonstrate that exercise intervention effectively alleviates behavioral abnormalities representative of ASD and MDD models, substantiating the notion that exercise holds promise as a complementary or disease-modifying treatment for psychiatric disorders. However, it is noteworthy that not all exercise regimes are effective. Exercise intensity emerges as a critical factor in regulating anxiety-like behaviors common in psychiatric disorders; however, the underlying molecular mechanisms remain elusive and warrant further investigation.

## Conclusions

Exercise showed promise as a complementary/disease-modifying treatment for ASD and MDD models, ameliorating its core symptoms. However, its impact on anxiety-like behaviors varied, possibly due to exercise intensity. Notably, LE was found to be optimal for alleviating the core symptoms and comorbid anxious behaviors in psychiatric conditions. While exercise holds potential, further research is needed to optimize its therapeutic use in ASD and MDD.

### Supplementary Information

Supplementary Material 1.

Supplementary Material 2.

## Data Availability

The datasets used and/or analyzed during the current study are available from the corresponding author upon reasonable request.

## Abbreviations

ASD: Autism spectrum disorder
EE: Energy expenditure
FST: Forced swim test
KO: Knockout
MDD: Major depressive disorder
ME: Moderate-intensity exercise
OFT: Open field test
RER: Respiratory exchange ratio
RST: Chronic restraint stress
TST: Tail suspension test
VT: Ventilation threshold
VWR: Voluntary wheel running
WT: Wild-type
LE: Low-intensity exercise
HE: High-intensity exercise
