# Supplementary material for: The influence of exercise intensity on comorbid anxious behavior in psychiatric conditions
Source: J Physiol Sci. 2024 Aug 2;74:39. doi: 10.1186/s12576-024-00930-7 (PMC11295499; doi:10.1186/s12576-024-00930-7)
Supplement: Supplementary file 1 — Supplementary Material 1. [file 12576_2024_930_MOESM1_ESM.docx]

**Supplementary Information for**

The influence of exercise intensity on comorbid anxious behavior in psychiatric conditions

Dong-Joo Hwang^a,b^, Tae-Kyung Kim^a,b,c, *^

^a^ Exercise Biochemistry Laboratory, Korea National Sport University, Seoul, Korea

^b^ Sport Science Institute, Korea National Sport University, Seoul, Korea

^c^ Department of Physical Education, Korea National Sport University, Seoul, Korea

**Corresponding author:** Tae-Kyung Kim, Ph.D.

Email: [spt21@knsu.ac.kr](mailto:spt21@knsu.ac.kr)

**This PDF file includes:**

• Supplementary Figure 1 (Supple Fig. 1)

• Supplementary Video 1

**
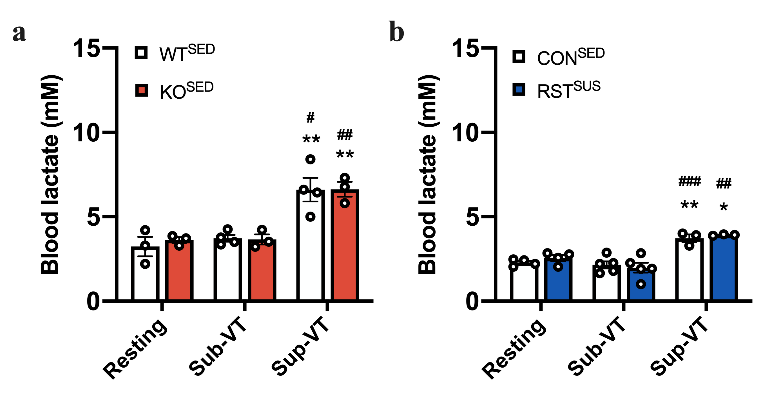
**

**Supple Fig. 1. Measurement of metabolic stress after a single bout of treadmill running with different intensities. a-b** The concentration of blood lactate after a single bout of treadmill running in WT^SED^, KO^SED^, CON^SED^, and SUS^SED^ mice (n = 3–5 mice/group). Sub-VT and supra-VT were defined as low- or light-intensity and high-intensity, respectively. Data are presented as mean ± SEM. Each circle represents individual data points. * p < .05, ** p < .01 versus resting, # p < .05, ## p < .01, ### p < .001 versus sub-VT.
